# Cubital Tunnel Syndrome Caused by a Synovial Cyst From the Ulnohumeral Joint

**DOI:** 10.7759/cureus.89017

**Published:** 2025-07-29

**Authors:** Alexis A Granados Flores, Arym P Preza Estrada, José Luis Villarreal Salgado, Gerardo S Rea-Martínez, Adrian Torres-Parlange

**Affiliations:** 1 Plastic and Reconstructive Surgery, Hospital Regional “Dr. Valentín Gómez Farías” del Instituto de Seguridad y Servicios Sociales de los Trabajadores del Estado (ISSSTE), Zapopan, MEX; 2 General Surgery, Hospital General Regional No. 66 del Instituto Mexicano del Seguro Social (IMSS), Juárez, MEX; 3 General Surgery, Hospital General “La Paz” del Instituto de Seguridad y Servicios Sociales de los Trabajadores del Estado (ISSSTE), La Paz, MEX

**Keywords:** articular cyst, cubital nerve, cubital neuropathy, elbow, plastic surgery

## Abstract

Compression neuropathies of the upper limb encompass a range of conditions in which nerve entrapment along its anatomical course results in clinical signs and symptoms that significantly impact patient quality of life and function. Early diagnosis and individualized treatment are essential elements of daily clinical practice. Depending on the nerve involved, various anatomical compression sites have been identified. In the case of the ulnar nerve, compression at the elbow may be caused by anatomical structures or joint-related pathological conditions, such as synovial cysts or vascular malformations. Although rare, these latter causes hold high academic value for both trainees and experienced plastic surgeons.

## Introduction

Cubital tunnel syndrome is the second most common entrapment neuropathy of the upper extremity, with an estimated prevalence of 2-6% in the United States. It results from compression of the ulnar nerve at the elbow and is characterized by sensory disturbances such as numbness and paresthesia, primarily in the ring and little fingers, as well as motor symptoms including hand weakness. In some cases, patients may also report localized pain at the elbow [1,2].

The ulnar nerve originates from the brachial plexus, specifically from the medial cord, which arises from the ventral rami of C8 and T1. It descends along the medial aspect of the arm, initially anterior to the intermuscular septum, then pierces it and continues through the cubital tunnel to enter the forearm [1]. Common compression sites, from proximal to distal, include the medial intermuscular septum, Struthers arcade, the cubital tunnel, and Osborne’s fascia. However, reports of compression due to joint volume increase, synovial cysts, and, less commonly, vascular structures are scarce [3].

The importance of early identification and diagnosis lies in providing prompt treatment to reduce the risk of potential complications, such as permanent nerve damage, hand deformity, loss of sensation and motor function, and impaired grip strength.

Physical examination

Typical clinical manifestations of cubital tunnel syndrome include paresthesia and numbness along the ulnar aspect of the forearm, the fourth and fifth digits. In chronic or severe cases, motor deficits may occur in the intrinsic hand muscles, leading to weakness and loss of fine finger coordination due to damage to motor fibers [4].

Several well-established clinical signs aid in the diagnosis. Froment’s sign is positive when the interphalangeal joint of the thumb flexes during a lateral pinch, indicating adductor pollicis weakness. Wartenberg’s sign is positive when the little finger remains abducted during attempted finger adduction, suggesting intrinsic muscle weakness. Tinel’s sign is considered positive when tapping over the cubital tunnel elicits paresthesia in the ulnar distribution. The elbow flexion test is positive when elbow flexion combined with shoulder abduction reproduces symptoms along the ulnar nerve path. These clinical findings strongly support the diagnosis of ulnar neuropathy and are integral to physical examination [5].

## Case presentation

A 68-year-old female with chronic degenerative conditions, including systemic arterial hypertension diagnosed six years ago and currently managed with losartan, as well as rheumatoid arthritis diagnosed 25 years ago and treated with mesalazine, acemetacin, and monthly golimumab with limited clinical response, presented to the hospital.

Her symptoms began in November 2023, with paresthesia and numbness in the ulnar aspect of the forearm and the fourth and fifth digits of the left hand. Electromyography (EMG) revealed severe axonal motor and sensory involvement of the ulnar nerve at the elbow (Table 1 and Table 2). Surgical decompression was indicated. Physical examination revealed joint deformities involving the interphalangeal and metacarpophalangeal joints bilaterally, as well as increased volume of the left humeroulnar joint.

**Table 1:** Motor nerve conduction study (left and right). Motor nerve conduction studies of the left ulnar nerve revealed a significant reduction in the amplitude of the motor response, with values of 0.2 mV at the elbow and 0.1 mV at the wrist. These findings were accompanied by a marked decrease in conduction velocity between the elbow and axilla (34.4 m/s) when compared to the contralateral right side, which showed normal conduction velocities ranging from 52 to 53 m/s. NCV, nerve conduction velocity

Nerve	Side	Site	Latency (ms)	Amplitude (mV)	NCV (m/s)	Distance (mm)	Segment
Median	Left	Wrist	4.0	3.1	64.2	170	Wrist-elbow
		Elbow	6.6	3.0	-	-	Elbow-axilla
Ulnar	Left	Wrist	12.2	0.1	5.9	80	Wrist
		Elbow	15.8	0.2	7.0	100	Wrist-elbow
		Axilla	23.7	0.1	34.4	100	Elbow-axilla
Median	Right	Wrist	4.3	5.9	69.6	190	Wrist-elbow
		Elbow	6.5	5.6	-	-	Elbow-axilla
Ulnar	Right	Wrist	2.8	1.6	49.7	190	Wrist-elbow
		Elbow	6.6	1.6	53.2	100	Elbow-axilla

**Table 2:** Sensory nerve conduction study. In the sensory nerve conduction study of the left ulnar nerve, no sensory responses were elicited, further supporting the presence of a conduction block or severe axonal involvement. NCV, nerve conduction velocity

Nerve	Side	Site	Latency (ms)	Amplitude (µV)	NCV (m/s)	Distance (mm)
Ulnar	Right	Wrist	3.7	243.3	37.6	140
Ulnar	Left	Wrist	NR	NR	-	140

Surgical management

**Figure 1:**
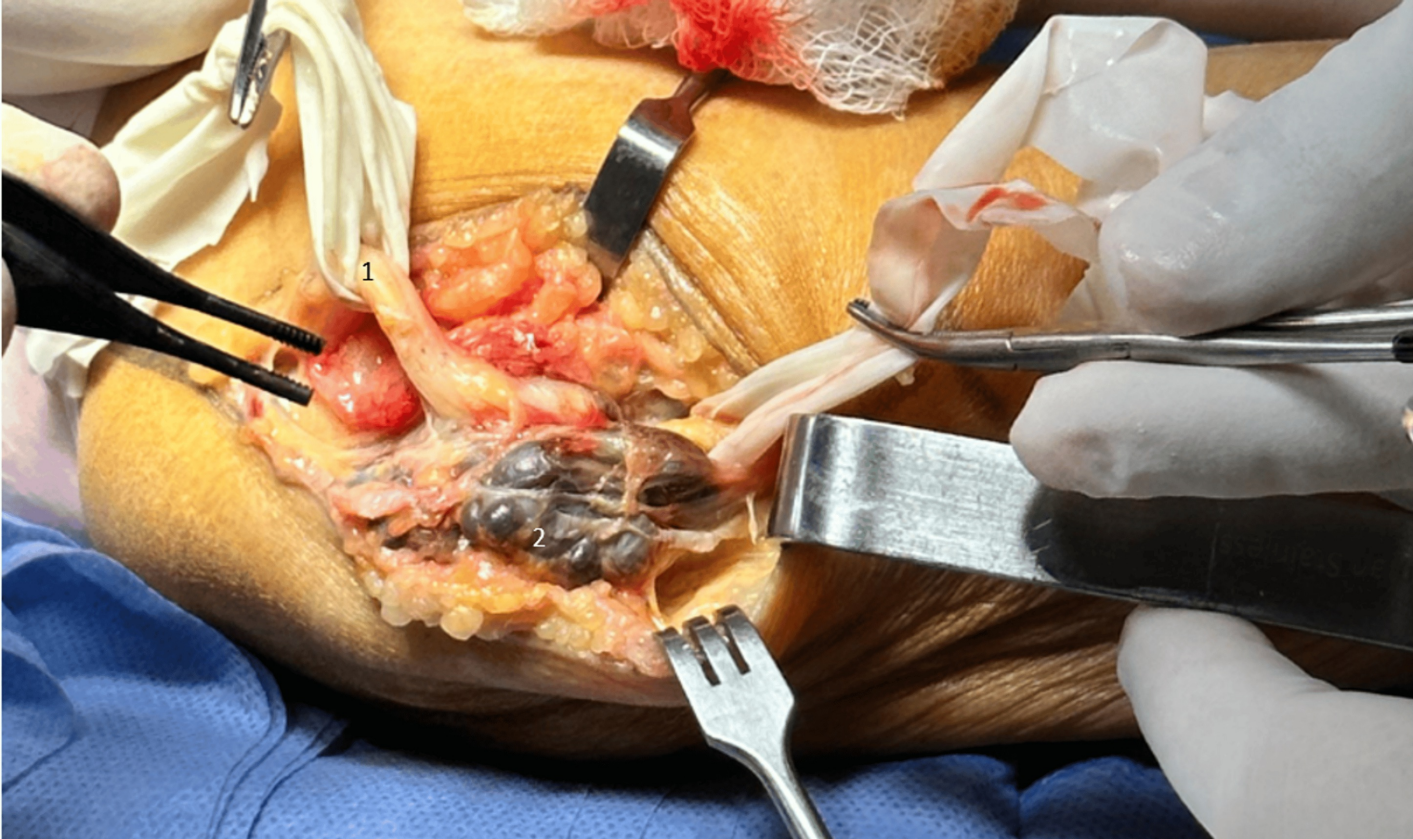
Synovial cyst closely associated with the ulnar nerve. 1 - ulnar nerve; 2 - synovial cyst

An open approach was selected due to its advantages in terms of reduced operative time and better visualization for complete cubital tunnel release. After aseptic preparation of the left upper limb and sterile field setup, a tourniquet was applied, and a 5 cm longitudinal incision was made along the course of the ulnar nerve at the medial epicondyle. Blunt dissection of soft tissues revealed a violaceous synovial cyst compressing the ulnar nerve (Figure 1).

**Figure 2:**
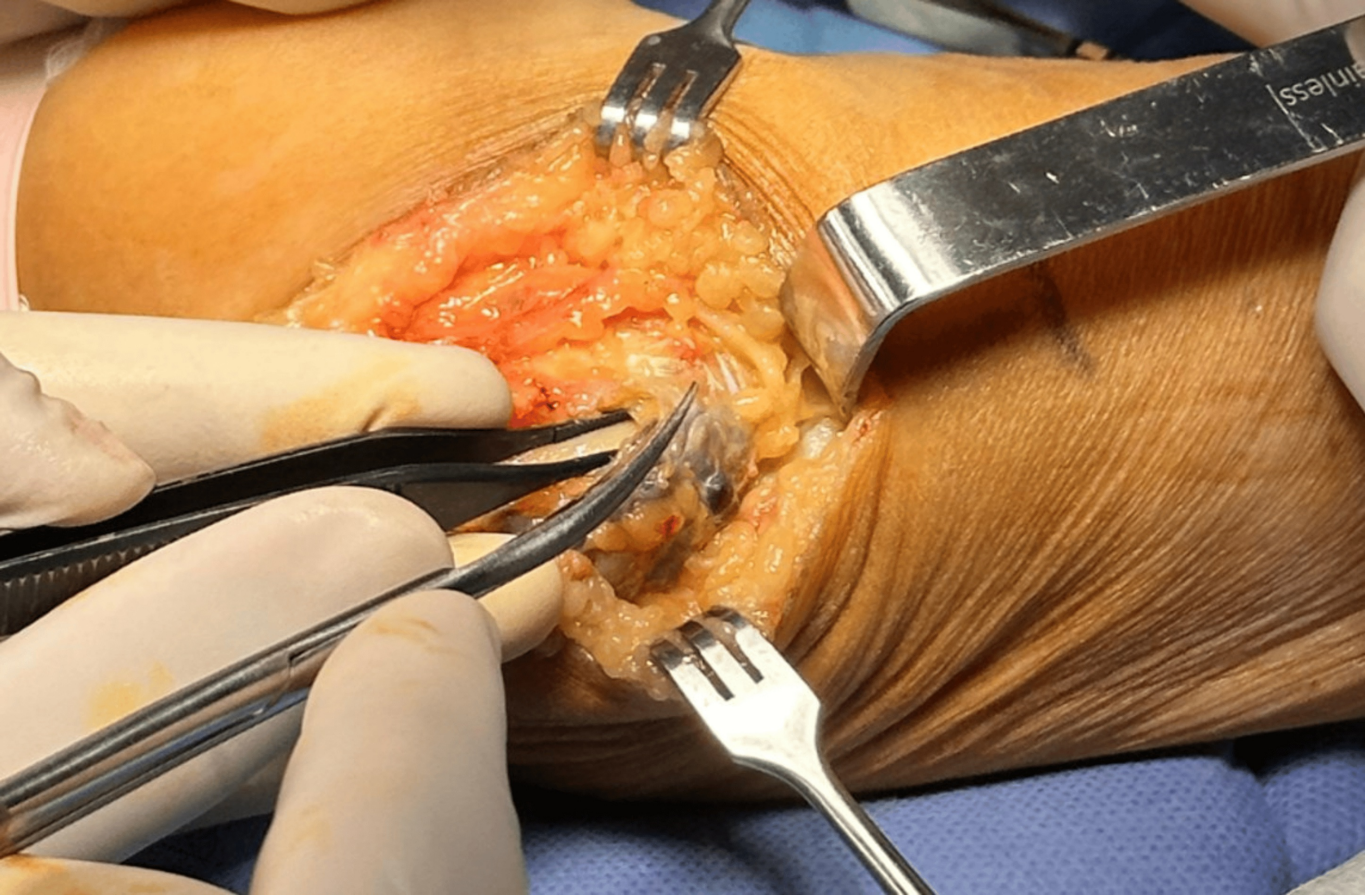
Cold dissection was performed to separate the synovial cyst from the ulnar nerve.

The cyst was carefully dissected and separated from the nerve. Upon opening, hemorrhagic synovial fluid was drained and sent for pathological analysis. Following cyst excision, a simple decompression was performed at Osborne’s ligament, and the surgical site was closed in layers using 4-0 Monocryl (absorbable) for soft tissues and 4-0 Nylon (non-absorbable) for the skin (Figure 2).

Postoperative course

Fourteen days after surgery, the patient presented for suture removal. Examination revealed proper wound healing and satisfactory closure of the surgical site. She reported near-complete resolution of numbness and paresthesia affecting the fingers and the ulnar aspect of the forearm. Sutures were removed without complications, and the patient was referred to the Department of Physical and Rehabilitation Medicine to begin physical therapy and rehabilitation.

## Discussion

The cause of compression in this case is rare compared to the common causes described in the literature. This underscores the importance of individualizing the diagnostic protocol for each patient, especially since this unusual finding is closely related to the patient’s preexisting rheumatologic condition. Previous reports have described cystic lesions as uncommon causes of ulnar nerve compression, often associated with underlying joint pathologies such as rheumatoid arthritis, which increases synovial proliferation and the risk of cyst formation [6,7].

The electrodiagnostic study confirmed severe mixed (axonal and demyelinating) ulnar neuropathy at the left elbow, affecting both motor and sensory fibers. Key findings included very low CMAP amplitudes (0.1-0.2 mV), absent sensory responses, and signs of active denervation with chronic reinnervation in intrinsic hand muscles. These results indicate a chronic focal lesion without proximal or generalized nerve involvement. According to AANEM guidelines, surgery is recommended in cases of severe axonal loss, persistent symptoms despite conservative treatment, and ongoing denervation on EMG. Surgical options include nerve decompression or anterior transposition, with excision of compressive lesions such as synovial cysts when present. Early surgery is essential to prevent irreversible muscle atrophy and improve functional recovery. In this case, the electrophysiological findings confirmed clinical suspicion and supported the surgical decision following established recommendations [8].

During surgery, once the synovial cyst was identified, exploration of the ulnar nerve toward Osborne’s ligament was also performed, as it is described as one of the four most common anatomical structures responsible for compression at the elbow. Open surgery allowed direct visualization of compressive zones and facilitated safe decompression. Endoscopic and minimal access techniques have shown favorable outcomes; however, open surgery remains the gold standard for addressing complex compressive lesions such as synovial cysts. Additional advantages of the open technique include reduced risk of nerve devascularization, lower anesthetic risk, and shorter operative time [9,10].

Treatment strategies vary widely and should be individualized based on the site of compression and patient-specific factors. Outcomes of open decompression are generally favorable, with studies reporting improvement in over 90% of treated patients [11]. Another key point is the potential for recurrence, which is higher when cysts are incompletely excised or when underlying inflammatory joint disease remains uncontrolled [12,13].

## Conclusions

This case highlights the critical importance of recognizing rare causes of cubital tunnel syndrome, particularly in patients with underlying inflammatory joint diseases such as rheumatoid arthritis. A high index of suspicion and individualized diagnostic evaluation, including detailed electromyography, are essential to accurately identify the severity and nature of ulnar nerve involvement and to guide timely surgical intervention. Surgeons should maintain vigilance for uncommon etiologies like synovial cysts compressing the ulnar nerve and adapt their surgical approach accordingly. Comprehensive intraoperative exploration of the cubital tunnel and adjacent anatomical structures is vital to identify all potential compression sites, ensuring effective decompression.

While less common, such etiologies must be considered by plastic and peripheral nerve surgeons during diagnosis and operative planning. Early and appropriate surgical management can prevent irreversible nerve damage, optimize functional recovery, and achieve favorable clinical outcomes, as demonstrated in this case.
